# Artificial Light at Night Affects Larval Growth Without Altering Survival or Pupation in Spongy Moth (*Lymantria dispar dispar*)

**DOI:** 10.1002/ece3.72311

**Published:** 2025-10-13

**Authors:** Nicola van Koppenhagen, Martin M. Gossner, Michael Eisenring, Doris Schneider Mathis, Jörg Haller, Janine Bolliger

**Affiliations:** ^1^ Swiss Federal Research Institute WSL Birmensdorf Switzerland; ^2^ Department of Environmental Systems Science, Institute of Terrestrial Ecosystems ETH Zurich Zurich Switzerland; ^3^ EKZ Zurich Switzerland; ^4^ Department of Geography University of Zürich Zurich Switzerland

**Keywords:** ALAN, ecological assessment, environmental pollution, insect development, insect pests, laboratory experiment, light color

## Abstract

Artificial light at night (ALAN) can disrupt circadian rhythms in nocturnal insects, but its effects on immature stages remain understudied. However, this knowledge is crucial, as a change in the development of insects can have ecological and economic consequences. For example, the spongy moth (*Lymantria dispar dispar*), native to Europe and Asia, has become an invasive species in North America, causing extensive defoliation of trees in forests and urban landscapes. Therefore, we investigated how LED light color influences larval development and pupation in the spongy moth. Larvae were reared under three light conditions simulating outdoor lighting: (1) neutral white LED (3700 K), (2) amber LED (2200 K), and (3) a dark control (no light). Results showed no significant differences in larval survival, growth patterns, or pupal stage outcomes between treatments. However, larvae exposed to 3700 K light reached significantly higher body masses at the eighth instar than those exposed to 2200 K and tended to be heavier than the control caterpillars. This is accompanied by a consistently higher weight gain at 3700 K in all larval stages. These results suggest that ALAN can influence larval growth—a crucial factor for fitness and population dynamics. Understanding the effects of ALAN on fitness‐related traits—especially in pest species like the spongy moth—is critical given the increasing prevalence of nighttime illumination.

## Graphical abstract

In this study, we investigated how artificial light at night (ALAN) affects larval development in the invasive spongy moth. Caterpillars exposed to 3700 K LED light reached higher body masses than those under 2200 K or darkness, suggesting ALAN may enhance growth and influence population dynamics. These results highlight the importance of considering the impacts of ALAN on fitness‐related traits, especially in pest species like the spongy moth.

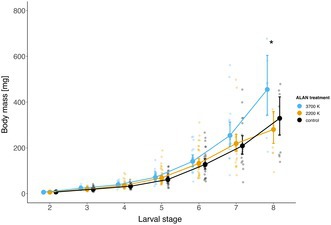

## 1 Introduction

Artificial light at night (ALAN) is a rapidly growing environmental pollutant and is increasingly recognized as a key driver of environmental change in the 21st century (Hölker et al. 2021). Nocturnal insects are particularly susceptible to ALAN, as many species are highly adapted to natural, low‐light night environments (Gaston et al. 2015; Owens et al. 2020; Owens and Lewis 2018; Van Langevelde et al. 2018). ALAN has been shown to interfere with circadian rhythms and temporal synchronization, resulting in altered activity patterns and behavioral desynchronization (Jiang et al. 2023; Levy et al. 2024). Specifically, exposure to ALAN impairs mating behaviors (Botha et al. 2017), reduces foraging efficiency (Van Langevelde et al. 2017), alters species interactions (Ceppi et al. 2025; Cieraad et al. 2023) and composition (Bolliger et al. 2022; Van Koppenhagen et al. 2024, 2025), and decreases fecundity (Honnen et al. 2019). Despite these well‐documented effects on adult insects, a critical knowledge gap remains regarding the impact of ALAN on earlier life stages, such as larvae and pupae, which may be equally or even more vulnerable to light pollution. Addressing this gap is essential for a comprehensive understanding of the ecological consequences of ALAN on insect populations.

In holometabolous insects, development is tightly regulated by environmental factors, with photoperiod and temperature playing critical roles (Saunders 2009). Natural day–night cycles are among the most reliable environmental cues available to organisms, and nearly all species rely on this diel pattern to regulate circadian systems and anticipate daily environmental changes (Gaston et al. 2017; Grubisic et al. 2019). By extending perceived daylength, ALAN can interfere with photoperiodic regulation and influence larval growth, development time, diapause induction, feeding activity, and metamorphosis (Haynes et al. 2023; Merckx et al. 2023; Schroer et al. 2019; Van De Schoot, Wesselingh, and Van Dyck 2025; Van Geffen et al. 2014). One of the earliest studies to examine the effects of different light colors of ALAN on moth development found that exposure to green or white light reduced maximum larval and pupal mass and led to an earlier onset of pupation (Van Geffen et al. 2014). Recent research has highlighted diverse and occasionally contradictory effects of ALAN on the development and survival of moth larvae. For example, Grenis and Murphy (2019) reported that exposure to ALAN in the nocturnal moth *Apamea sordens* (Lepidoptera: Noctuidae) led to a slower growth rate, prolonged development, and a lower body mass. Similarly, ALAN accelerated larval development and pupal duration but resulted in lower pupal mass and increased larval mortality in the garden tiger moth ( *Arctia caja* , Lepidoptera: Erebidae) (Van De Schoot, Lonnoy, et al. 2025). However, not all studies have found significant effects of ALAN on developmental traits. Haynes et al. (2023) observed neither an influence of ALAN on the development time from larvae to pupae nor the pupal mass in the monarch butterfly (*Danaus plexippus*, Lepidoptera: Nymphalidae). Despite these few case studies, larval and pupal responses to ALAN remain underexplored, particularly under controlled conditions and with commercially relevant lighting technologies. Addressing these knowledge gaps is critical to fully understanding the consequences of ALAN, as developmental impacts can lead to broader ecological or ecosystem consequences, many of which remain poorly understood (Grubisic and Van Grunsven 2021; Owens et al. 2020; Sanders and Gaston 2018).

In this study, we conducted a controlled laboratory experiment to test how different LED streetlight spectra affect the larval development and pupation of the spongy moth *Lymantria dispar dispar* (Lepidoptera: Erebidae), an economically important species that has caused severe damage to trees in North America and Europe (Rindos and Liebhold 2023). This species has nocturnally active larvae that undergo multiple molts before pupation. From the second instar to adult emergence, individuals were reared under three nighttime light conditions: (1) neutral white LED light (3700 K), (2) amber LED light (2200 K), and (3) a dark control with no nighttime lighting. The LED treatments represent commercially available streetlight spectra currently used in outdoor lighting. Based on a previous study (Van De Schoot, Wesselingh, and Van Dyck 2025), we hypothesized that caterpillars exposed to ALAN will experience higher mortality rates compared to those raised in darkness, potentially due to increased stress or disrupted circadian rhythms (H1). In line with previous studies performed on nocturnally active caterpillars (Grenis and Murphy 2019; Van Geffen et al. 2014), we also expected that ALAN will reduce larval weight gain over time, reflecting slower growth rates, which in turn should result in lower maximum larval and pupal masses (H2). We further anticipate, in line with for example, Van De Schoot, Lonnoy, et al. (2025) and Van Geffen et al. (2014), that overall development will be faster when exposed to ALAN, with caterpillars taking less time to reach pupation and experiencing accelerated pupal durations (H3). By quantifying different traits, our study investigates whether exposure to ALAN—and the spectral characteristics of that light—affects the growth, development, and survival of nocturnal moth larvae.

## 2 Methods

### 2.1 Study Species

The spongy moth (*L. dispar dispar*, Lepidoptera: Erebidae) is an invasive, univoltine nocturnal moth native to much of the Palearctic region and introduced to eastern North America. It is a notorious forest pest with an exceptionally broad host range. In many populations, larvae exhibit a characteristic diel movement—ascending trees at night to feed on leaves and descending in the morning to seek shelter in bark crevices or under rocks. Under certain conditions, populations can grow explosively and increase by several orders of magnitude within a few years. During outbreak events, they can completely defoliate forests, which not only suppresses tree growth but may also disrupt water balance and lead to increased tree mortality (Rindos and Liebhold 2023). The spongy moth has a well‐documented life cycle (Boukouvala et al. 2022; Ponomarev et al. 2023), making it an ideal model species to rear and study the impacts of ALAN in a controlled laboratory setting.

### 2.2 Laboratory Set‐Up and Light Treatments

The study was conducted in two climate chambers (3.8 m × 3.6 m × 2.15 m; L × W × H) automatically programmed to maintain a constant temperature of 26°C and 70% relative humidity. To further monitor for any major deviations from the desired climate settings, three data loggers (EasyLog EL‐USB‐2, Lascar Electronics) were placed in each chamber and checked regularly. In each climate chamber, three tables (1.6 m × 0.6 m × 0.75 m; L × W × H) were arranged, one per treatment. Caterpillars were exposed to three nighttime light treatments, which consisted of two different color temperatures: neutral white (3700 K) and amber (2200 K) (400 ± 40 lx measured in the middle of the table; see spectral distribution in Figure S1A), as well as a dark control without any light present at night. The 400 lx treatment represents a deliberately high illumination level, chosen to test whether very strong ALAN exposure can lead to measurable responses. To prevent light interference between the treatments, light‐impermeable tarpaulins (anthracite, 2 m × 3 m, 270 g/m^2^; kellerfahnen.ch) were installed between the tables. For the nighttime light treatments, we employed state‐of‐the‐art LED streetlight luminaires (Izylum 1, Schréder Swiss AG). We therefore installed two streetlights on aluminum poles (1.7 m) in each climate chamber, which were secured into a wooden base for stability. The control treatment remained dark (to see the experimental setup, see Figure S2). Each streetlight setup was positioned at the center of the longer side of its corresponding table, placing the head directly above the midpoint. The luminous flux of all streetlight luminaires was standardized to 1500 lm to ensure consistency across treatments, with calibration conducted by the Swiss Metrological Institute METAS in Bern (Table S1). During the day, all caterpillars received the same light treatment. To simulate daylight conditions during the day, we used linear LED lights (SANlight FLEX II‐25; see spectral distribution in Figure S1B). These linear LED lights were mounted on the ceiling to ensure a uniform height across all tables. The daylight LEDs were installed at the same height as the streetlights and centered above each table to ensure even illumination and to avoid any shadowing effects caused by the streetlight heads during the night (Figure S2). All treatments received 15 h of simulated daylight per 24‐h period, from 06:00 to 21:00. During the remaining 9‐h night period, the control treatment remained dark (15:9 h light:dark), whereas the two light treatments received continuous illumination from the streetlights throughout the entire night phase (15:9 h light:ALAN). The switching between the lights for all the treatments was automatically regulated by a timer. The installation and maintenance of the LED streetlights were carried out by technical experts from the Electricity Supplies of the Canton of Zurich (EKZ), ensuring consistent and uninterrupted application of the light treatments throughout the study.

### 2.3 Caterpillar Handling

The spongy moth caterpillars used in this experiment originated from egg masses of a multi‐generational population raised under a natural day‐night cycle in a controlled laboratory setting in Munich, Germany (Bayerische Landesanstalt für Wald und Forstwirtschaft LWF). For our study, approximately 1500 eggs were randomly selected and distributed into cylindrical containers (40 mm height × 86 mm diameter). The eggs were kept under control treatment conditions. Upon hatching, the caterpillars were reared in groups of ~50 individuals and fed an artificial diet (Frontier Agricultural Science, F9630B). Once the caterpillars reached the second instar, 50 individuals were randomly assigned to each treatment. This procedure was carried out in both climate chambers, resulting in 100 caterpillars per treatment and a total of 600 caterpillars. At this stage, they were transferred to individual containers (40 mm height × 86 mm diameter) closed with a white fine‐mesh fiberglass fabric. All containers contained a cube of artificial diet (2 cm × 2 cm × 2 cm). The cube was replaced weekly to prevent it from drying out. Furthermore, the caterpillar containers were evenly distributed across four equally sized sectors marked on the tables (Figure S2). Because light intensity decreases towards the edges of the table (to about a third of the measured intensity in the middle of the table) and microclimatic variation (e.g., humidity, temperature) might occur within the climate chambers, the containers were regularly rotated among these sectors within each treatment to ensure comparable conditions and minimize potential spatial effects. The rotation was performed weekly after the diet replacement. The individual caterpillar weighing started once the caterpillars reached the second instar (upon transfer to individual containers) and then 48h after each subsequent instar change. Weighing was conducted using a precision balance (ME403, Mettler Toledo, ±1 mg), with caterpillars gently transferred onto a pre‐weighed weighing boat (see Figure S3). The experiment ran from 21 February to 12 June 2024, totaling 112 days.

### 2.4 Statistical Analysis

All statistical analyses were performed in R version 4.2.2 (R Core Team, 2022). Linear mixed‐effect models (LMM) were fitted using the lme4 package (Bates et al. 2015; version 1.1.30) to assess the effects of the two ALAN treatments and the dark control on the body mass of caterpillars. The model included the fixed effects of treatment (3700 K, 2200 K, dark control), instar stage (ordered factor), the treatment × instar interaction, and the climate chamber, with individual caterpillars included as a random effect to account for repeated measures. Body mass was log‐transformed to improve normality and homoscedasticity of residuals. To test for differences between treatments at each larval stage, we estimated marginal means of body mass for each treatment within each larval instar using the emmeans package (Lenth 2025; version 1.8.0). We then performed a Post hoc analysis consisting of pairwise comparisons between treatments within each instar level using the contrast() function of the emmeans package (method = “pairwise”) with *p*‐values adjusted for repeated measures using the False Discovery Rate (FDR) method (adjust = “fdr”). Pupation measurements, including pupation mass, pupation duration, and total developmental time to pupation, were analyzed using linear models (LM) fitted by ordinary least squares with Gaussian errors. Each response variable (pupation mass, pupation duration, and total development time) was log‐transformed to improve normality and homoscedasticity of residuals. The fixed effects included treatment, climate chamber, and the number of larval stages completed before pupation. The explanatory variables entering the models were checked for overall model performance using the DHARMa package (Hartig et al. 2024; version 0.4.6). Model performance was further assessed using R2 from the MuMIn package (Bartoń 2024, version 1.47.5).

To test treatment effects on mortality, we used a Cox proportional hazards model using coxph() from the survival package (Therneau 2024; version 3.4.0) with time to death and death status (1 = died, 0 = survived until pupation) as the response, and treatment and climate chamber as predictors. A separate Cox model was fitted for pupation timing, using time to pupation and pupation status (1 = pupated, 0 = did not pupate) as the response. This model included treatment, climate chamber, and the number of larval stages completed before pupation as predictors. Kaplan–Meier survival curves were generated with the survfit() function from the survival package based on survival objects created using the surv() function, and plotted using ggsurvplot() from the survminer package (Kassambara et al. 2024; version 0.5.0).

## 3 Results

### 3.1 Larval Mortality

**FIGURE 1:**
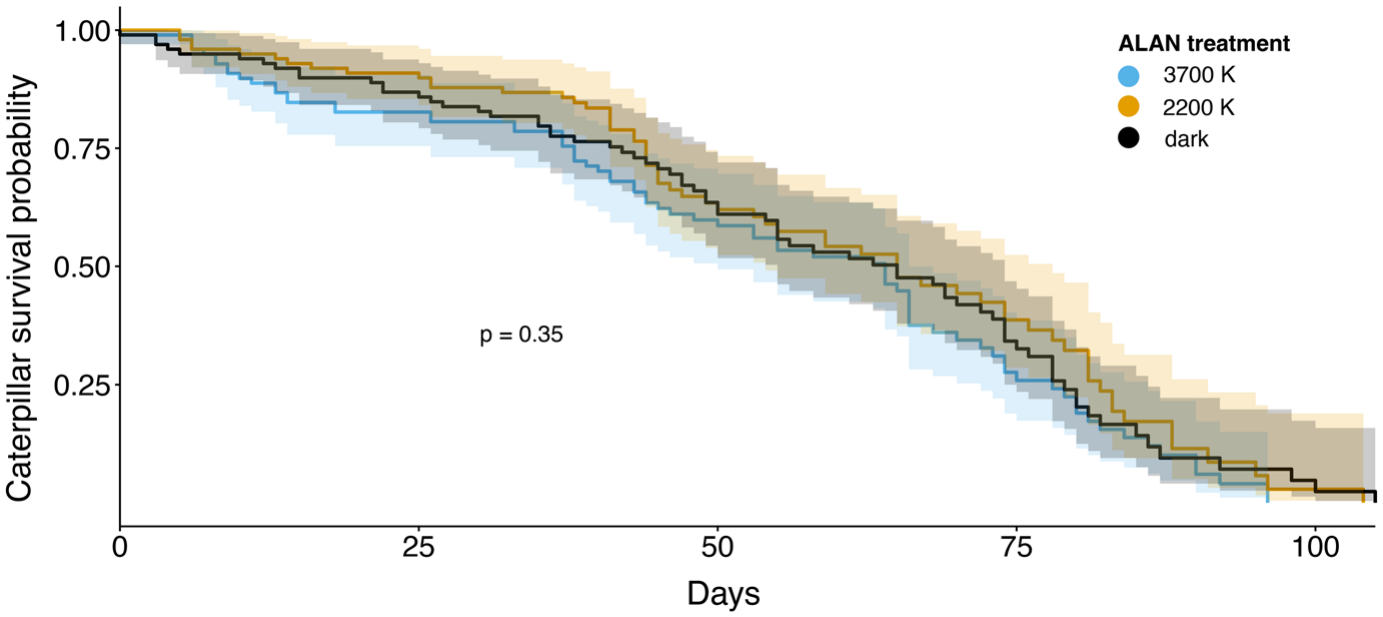
Kaplan–Meier survival curves for spongy moth caterpillars that died before pupation (*Lymantria dispar dispar*) under three ALAN treatments (3700 K, 2200 K, and dark). Survival probabilities are shown relative to the subset of individuals that died, excluding the ones who survived to pupation. The shaded band represents the 95% confidence interval.

No significant differences in caterpillar mortality were observed between the light treatments over the entire experimental period of 112 days (*χ* ^2^ = 2.10, *p* = 0.35; Figure 1). The proportion of caterpillars that survived to pupation was similar across treatments, with 75% mortality in the 3700 K group, 63% in the 2200 K group, and 72% in the control. For detailed regression results, see the Table S2.

### 3.2 Body Mass

**FIGURE 2:**
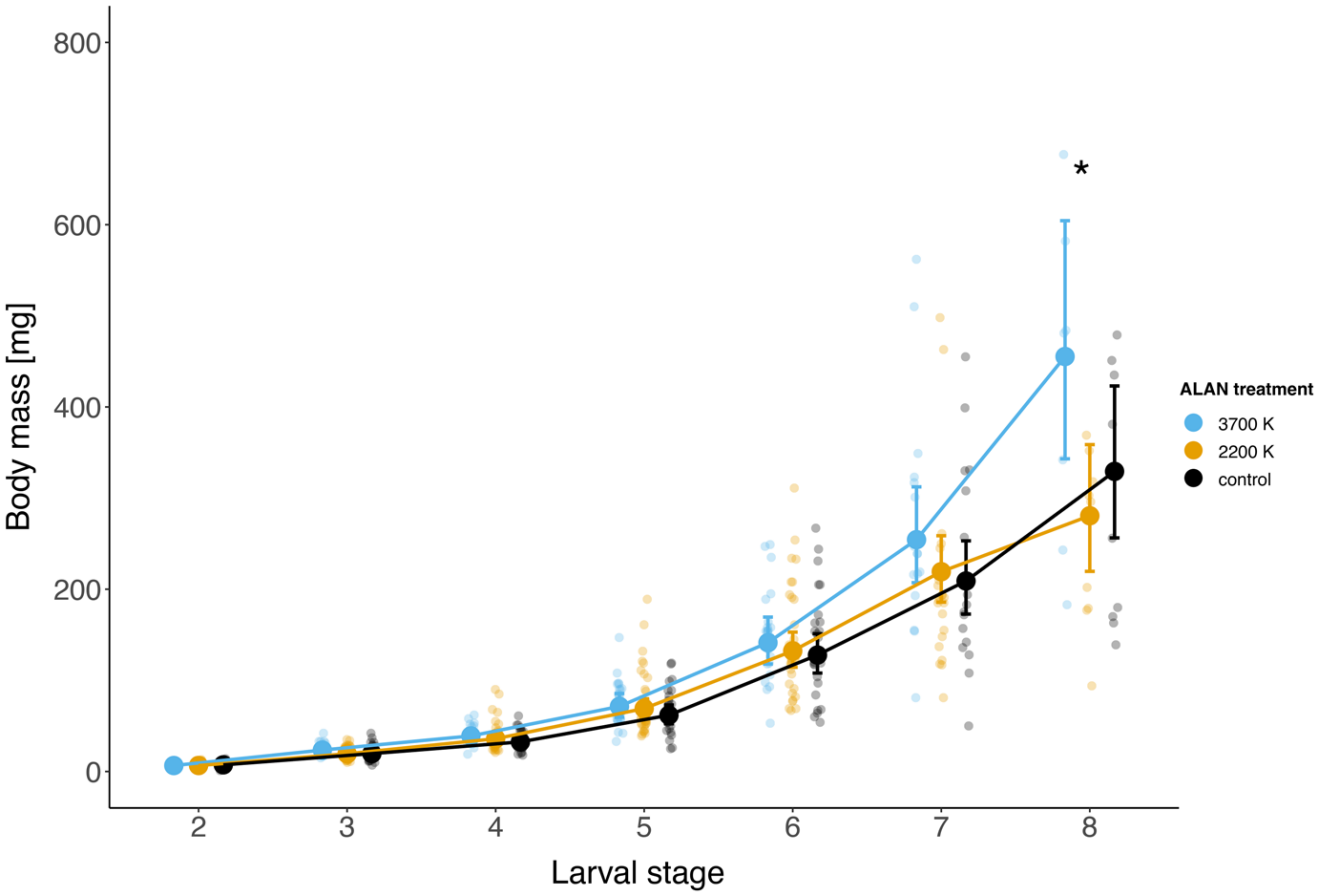
Estimated mean body mass (±95% confidence intervals) of spongy moth (*Lymantria dispar dispar*) larvae across different instars (stages 2–8) under three artificial lights at night (ALAN) treatments: 3700 K, 2200 K, and a dark control. Asterisks indicate significant differences between treatments *p* < 0.05 within a larval stage. Small, more transparent dots show body mass of individual caterpillars.

Caterpillar body mass, measured across larval stages 2–8, increased consistently over time, with no statistically significant differences among treatments at most stages. However, caterpillars exposed to 3700 K light reached significantly higher mean body mass at larval stage 8 compared to those exposed to 2200 K (3700 K: 442 mg vs. 2200 K: 287 mg; *t*(479) = 2.54, *p* = 0.03), while not differing significantly from the dark control but also showing a clear trend towards a higher body mass at this stage. This suggests a potential influence of cooler color temperatures on late‐stage growth, with a general trend of higher mass across all larval stages in the 3700 K group (Figure 2). When considering the final recorded larval mass of all individuals that pupated, however, no statistically significant differences among treatments were detected, although a trend towards higher body mass in the 3700 K group remained (Figure S4). For detailed regression results, see the Tables S3–S7.

### 3.3 Pupation

**FIGURE 3:**
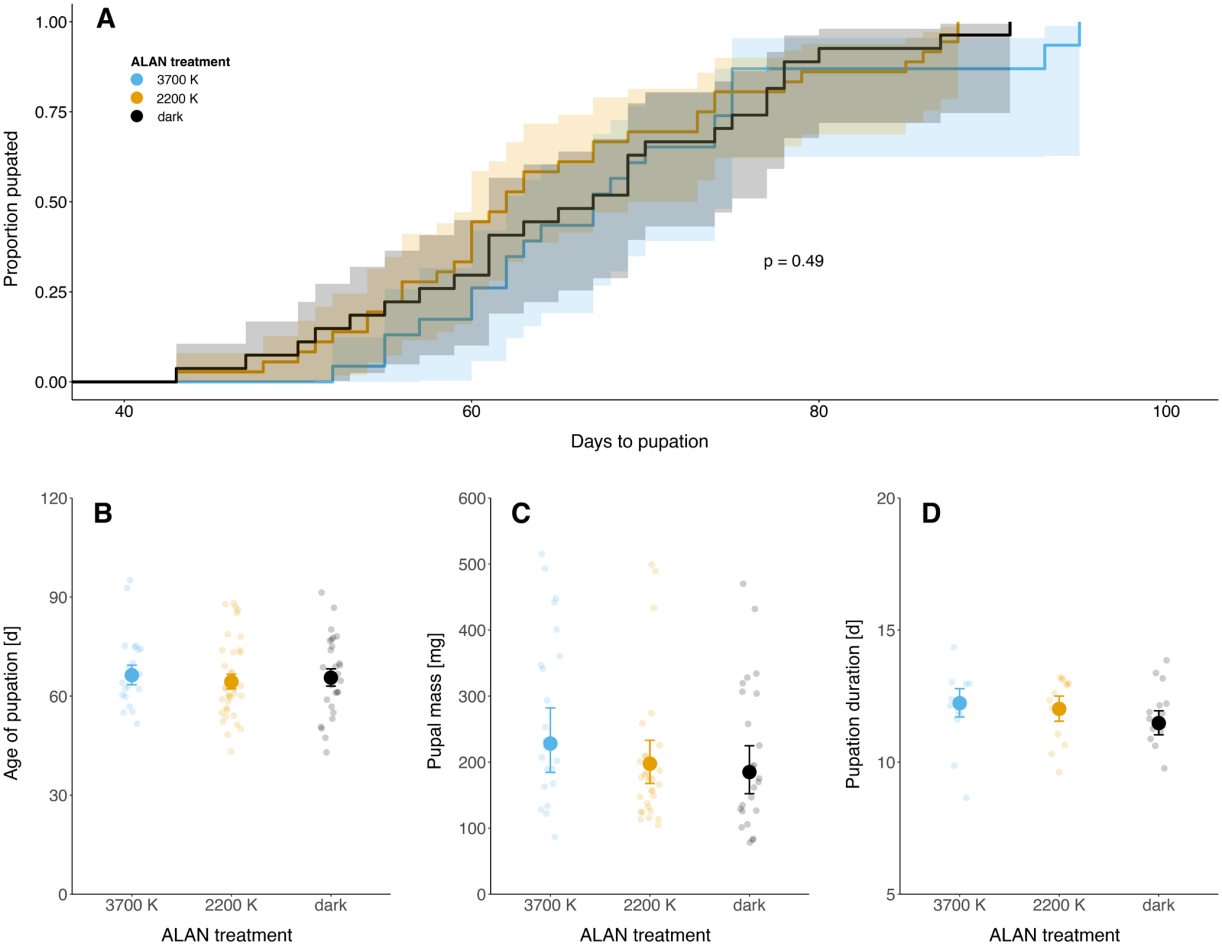
Pupation outcomes of spongy moth (*Lymantria dispar dispar*) caterpillars across three artificial light at night (ALAN) treatments (3700 K, 2200 K, and dark control). (A) Proportion pupated over time, (B) age at pupation, (C) pupal mass, and (D) pupation duration. Large dots represent estimated mean values (±95% confidence intervals); small, more transparent dots represent individual caterpillar values.

There were no significant effects of ALAN on any measured pupation parameters. The cumulative proportion of caterpillars that pupated over time was consistent across all treatments (*χ* ^2^ = 1.45, *p* = 0.49), with most individuals in each group completing pupation within a comparable time frame of 43–95 days. However, we observed a slight delay in the onset of pupation in the 3700 K group, and it also took them longer until all the caterpillars reached pupation compared to the other treatments (Figure 3A). This is also reflected in a slightly later, albeit statistically insignificant, mean pupation age in the 3700 K group (Figure 3B). Pupal mass did not differ significantly between treatments, although caterpillars exposed to ALAN tended to have slightly higher masses (3700 K: 228 mg, +23% vs. control; 2200 K: 198 mg, +7% vs. control; Figure 3C). Similarly, the duration of the pupal stage did not show any significant difference between the treatments (Figure 3D). For detailed regression results, see the Tables S8–S14.

## 4 Discussion

Our study examined the effects of ALAN—using commercially available LED streetlights with two color temperatures (2200 K, 3700 K)—on the development of the economically important forest moth *L. dispar dispar*. Caterpillars exposed to cooler 3700 K LEDs—a color temperature frequently found in urban environments, particularly in public spaces and modern LED street lighting—reached significantly higher body masses at the latest instar stage compared to those reared under warmer 2200 K lights and a clear trend towards a higher body mass compared to the control. This result is accompanied by a broader trend of increased weight gain at 3700 K across all larval stages. However, we found no statistically significant effects of LED color on mortality, pupal mass, age at pupation, or pupal duration. Our findings suggest that while ALAN does not strongly affect overall life‐history parameters, it can nonetheless influence key growth traits during the larval stage, potentially impacting population dynamics in this pest species.

Survival rates were broadly similar across treatments, with no statistically significant differences in overall mortality. This is in contrast to our first hypothesis (H1), stating higher caterpillar mortality when exposed to ALAN compared to the dark control, based on recent findings by Van De Schoot, Lonnoy, et al. (2025). The absence of mortality effects underlines that ALAN effects were subtle, which is consistent with a study of Grenis and Murphy (2019), where they also observed no impact of light treatment on larval survival. In contrast to our second hypothesis (H2), which predicted lower body masses in caterpillars exposed to ALAN due to potentially reduced growth rates, we observed a clear trend of higher body masses across all larval stages exposed to 3700 K light. This contrasts with previous studies on other nocturnal moth species, such as *A. sordens* (Grenis and Murphy 2019) and *Mamestra brassicae* (Van Geffen et al. 2014), which reported slower growth rates and reduced body mass under ALAN. Specifically, Van Geffen et al. (2014) found that caterpillars exposed to white light reached lower body masses than those exposed to red light, while our results indicate the opposite effect, with colder light supporting higher growth rates than warmer color temperatures. This unexpected positive effect of 3700 K ALAN on caterpillar growth is surprising, given that spongy moth caterpillars are predominantly nocturnal (the first three instars are thought to be diurnal). One would expect that certain light spectra that are closer to our daylight treatment—such as 3700 K (which is more similar to our daylight treatment than 2200 K)—suppress their natural feeding activity (see spectral distribution of all the treatments in the Figures S2 and S3). It is possible that differences in 3700 K and daylight regimes were subtle enough that caterpillars were not able to differentiate between day and night based on light cues. As a result, caterpillars may have adjusted their feeding patterns independent of light cues, which may have led to increased feeding activities during the night. In contrast, the greater spectral difference to the daylight treatment under 2200 K light may have preserved day–night distinctions better than in 3700 K, resulting in growth patterns more similar to the dark control. Overall, our findings highlight the need for detailed behavioral observations under different light settings to better understand how ALAN influences feeding rhythms and activity patterns in nocturnal caterpillars. Our third hypothesis (H3), which states that ALAN causes the caterpillar to develop faster and take less time to reach pupation, was also not confirmed. We found no significant effect of ALAN on the timing or duration of pupation, in contrast to studies that found an earlier onset of pupation (Van Geffen et al. 2014) as well as a shorter pupation duration (Van De Schoot, Wesselingh, and Van Dyck 2025; Van Geffen et al. 2014). Despite a slight, though statistically insignificant, delay in the onset of pupation for caterpillars exposed to 3700 K light, the overall timing and duration of pupation were consistent across treatments, suggesting that the effects of ALAN on development duration in this experiment were minimal. Notably, the observed slight delay in larval development under the 3700 K treatment may help explain the higher body mass at the eighth instar, as slower development often allows for increased growth before pupation.

Taken together, our findings suggest that although we did not find a significant change in developmental timing, we did detect some influence of ALAN on larval growth patterns. One possible reason for the limited overall effects and the differences from previous studies could be the use of a long‐term laboratory population, which may differ in sensitivity to our treatments compared to wild populations. However, since these individuals were kept under a simulated natural day‐night cycle and had never been exposed to ALAN before, strong adaptation to such conditions seems unlikely. Still, the observed increase in larval mass when exposed to 3700 K light is noteworthy given that insect body mass is widely accepted as a proxy for lifetime fitness, as it directly influences reproductive output and overall survival (Beukeboom 2018). Heavier larvae are often expected to pupate earlier, as they reach the critical weight threshold for metamorphosis faster (Price et al. 2011; Schoonhoven et al. 2005). However, in our experiment, despite observing a trend towards higher larval mass when exposed to 3700 K ALAN, we found no corresponding acceleration in pupation timing. This suggests that factors beyond mass, such as hormonal regulation or energy allocation, might play a more significant role in determining pupation age in spongy moths and therefore need more attention in future studies. It should also be noted that larval mass does not necessarily translate into adult mass; variation in water loss during pupal development—often sex‐specific—could further alter adult body size (Molleman et al. 2011; Van De Schoot, Lonnoy, et al. 2025). Nevertheless, the larger body size observed at late larval stages under 3700 K could have critical implications for adult fitness, particularly since lights of this or similar color temperature are widely used in modern urban and suburban outdoor lighting. In capital breeders like spongy moths, mass gained as larvae is particularly important, as unlike income breeders they do not feed as adults. As a result, the energy acquired during larval development is the only reserve available for adult moths to find a mate and reproduce (Jervis et al. 2005). This could translate into increased fecundity, as larger females typically produce more eggs (Loewy et al. 2013), potentially amplifying population growth in potential pest species. Moreover, larger males might gain advantages in flight endurance, longevity, and sperm competition (Iyengar and Eisner 1999). Though our observed mass differences compared to the control were not statistically significant, the trend suggests that even small shifts in larval growth when exposed to ALAN could contribute to accelerating mass outbreaks in spongy moth populations, with potentially far‐reaching ecological and economic consequences. A limitation of our study is that sex was not determined, although *L. dispar dispar* shows strong sexual size dimorphism (Boukouvala et al. 2022). However, sex is genetically determined shortly after oviposition (Moronuki et al. 2025), and larvae were randomly assigned to treatments, which reduces the likelihood of systematic sex ratio differences affecting our results. Furthermore, future studies should not only investigate laboratory‐reared individuals but also include wild populations to determine whether these findings hold under natural field conditions and under less “extreme” lighting conditions (< 400 lx). While 400 lx is higher than most encountered streetlight intensities, such values can easily occur on foliage close to streetlights. We therefore used this “extreme” treatment as a first step to test for potential effects, with the expectation that follow‐up studies will address more ecologically typical conditions.

Overall, our results provide novel evidence that ALAN can influence the development of nocturnal Lepidoptera species, with potential consequences for both ecosystem functioning and pest dynamics. At the same time, our study also highlights the need for further research into the species‐specific effects of ALAN, as well as the broader ecological and economic risks associated with light pollution. A deeper understanding of the full range of potential ALAN impacts on the growth, fitness, and population dynamics of insects in general and specifically pest species like the spongy moth will be essential for developing effective conservation and pest management strategies in an increasingly illuminated world.

## Author Contributions

**Nicola van Koppenhagen:** conceptualization (lead), data curation (lead), formal analysis (lead), investigation (lead), methodology (lead), project administration (equal), visualization (lead), writing – original draft (lead). **Martin M. Gossner:** conceptualization (supporting), formal analysis (supporting), funding acquisition (supporting), methodology (equal), resources (equal), validation (equal), writing – review and editing (equal). **Michael Eisenring:** formal analysis (equal), methodology (supporting), writing – review and editing (equal). **Doris Schneider Mathis:** conceptualization (equal), investigation (equal), methodology (supporting), writing – review and editing (supporting). **Jörg Haller:** funding acquisition (supporting), resources (lead), validation (equal), writing – review and editing (supporting). **Janine Bolliger:** conceptualization (equal), funding acquisition (lead), methodology (equal), project administration (lead), resources (equal), supervision (lead), validation (equal), writing – review and editing (equal).

## Conflicts of Interest

The authors declare no conflicts of interest.

## Supporting information


**Appendix S1:** ece372311‐sup‐0001‐AppendixS1.docx.

**Appendix S2:** ece372311‐sup‐0002‐AppendixS2.docx.

## Data Availability

The data supporting the findings of this study can be freely accessed at Figshare: https://doi.org/10.6084/m9.figshare.29341487.
