# Supplementary material for: Artificial Light at Night Affects Larval Growth Without Altering Survival or Pupation in Spongy Moth (Lymantria dispar dispar)
Source: Ecol Evol. 2025 Oct 13;15(10):e72311. doi: 10.1002/ece3.72311 (PMC12517357; doi:10.1002/ece3.72311)
Supplement: Supplementary file 1 — Appendix S1: ece372311‐sup‐0001‐AppendixS1.docx. [file ECE3-15-e72311-s002.docx]

**Supplementary materials**

**Supplementary Figures**

**Figure S1A.** Spectral distribution covered by the two LED colors: 3700 K (top), and 2200 K (bottom).


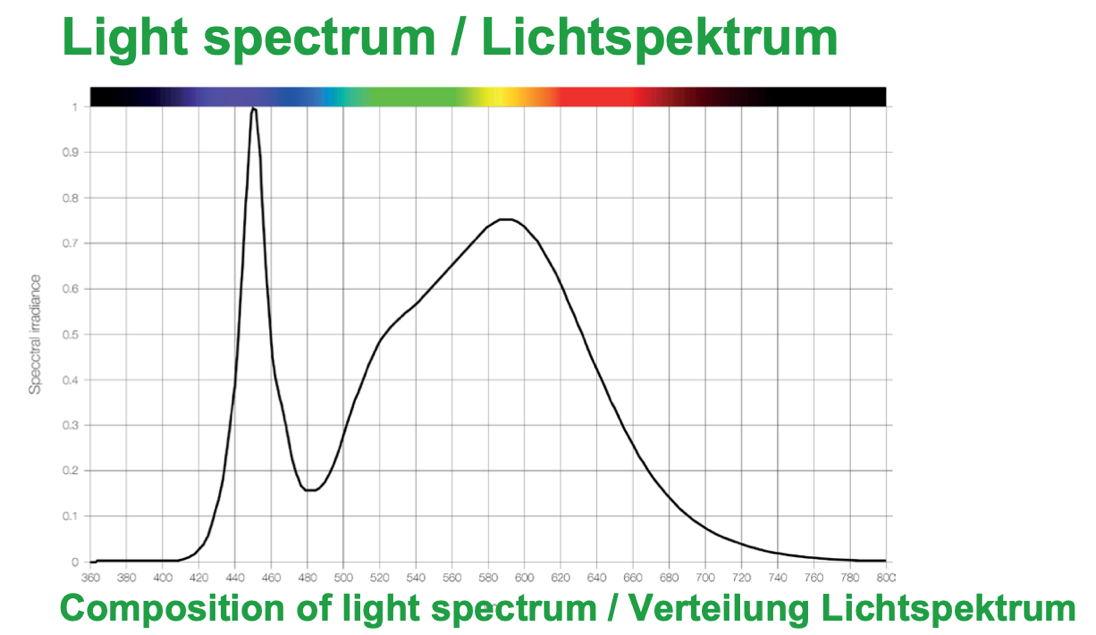


**Figure S1B.** Spectral distribution covered by the simulated daylight LED (Source: https://www.sanlight.com/wp-content/uploads/2024/02/datenblatt-flex-ii-a4-07-de-en.pdf).

**Figure S2.** Experimental setup inside the climate chambers. Three treatment tables separated by light-impermeable tarpaulins to prevent light spillover between the treatments. Each table was subdivided into four sectors for even distribution and rotation of caterpillar containers. Two LED streetlights and LED for simulated daylight were placed in the climate chamber for the different treatments. Note that we had to crop together two photos because we were not able to get the whole set up in one shot, therefore the slight change in perspective in the control treatment.

**Figure S3.** Weighing setup used to measure caterpillar mass. (A) Caterpillars were gently transferred onto a pre-weighed weighing boat and then (B) placed on a precision balance (ME403, Mettler Toledo; accuracy ±1 mg).

**Figure S4.** Estimated mean body mass (± 95 % confidence intervals) of spongy moth (Lymantria dispar dispar) of the last larval instar before pupation under three artificial light at night (ALAN) treatments: 3700 K, 2200 K, and a dark control. Small, more transparent dots show body mass of individual caterpillars.
