# Supplementary material for: Artificial Light at Night Affects Larval Growth Without Altering Survival or Pupation in Spongy Moth (Lymantria dispar dispar)
Source: Ecol Evol. 2025 Oct 13;15(10):e72311. doi: 10.1002/ece3.72311 (PMC12517357; doi:10.1002/ece3.72311)
Supplement: Supplementary file 2 — Appendix S2: ece372311‐sup‐0002‐AppendixS2.docx. [file ECE3-15-e72311-s001.docx]

**Supplementary Materials**

**Supplementary tables**

**Table S1.** Light technical information for all light treatments (LED color, dimming levels and luminaire shapes). Correction factors were applied to standardize the luminous flux for all light treatments (luminaires with and without diffusors, all dim levels, and light colors). All luminaires were set to a luminous flux of 1500 lumens.

| **Luminaire model** |  |  |
| --- | --- | --- |
| Izylum1, 20 LED with lens optics |  |  |
|  |  |  |
| **Light color temperature (Kelvin)** |  |  |
| **Color description** | **Manufacturer specification** | **Lab measurement** |
| Amber | 2200 K | 2155 K |
| Neutral white | 4000 K | 3699 K |
|  |  |  |
| **Luminous flux (Lumen)** |  |  |
| **Color description** | **Lab measurement** | **Experiment (with corrections)** |
| Amber | 2231 lm | 1500 lm |
| Neutral white | 1938 lm | 1500 lm |

**Table S2.** Cox proportional hazard results for caterpillar mortality (survival analysis). The explanatory variables are ALAN treatment (three levels) and climate chamber (two levels). The model estimates hazard ratios based on treatment and chamber effects on larval mortality.

Statistical levels of significance: *** < 0.001; ** < 0.01; * < 0.05.

| *Term* | *Coefficent* | *Hazard ratio* | *95% CI* | *p-value* |
| --- | --- | --- | --- | --- |
| 2200 K vs 3700 K | -0.24 | 0.79 | [-0.57, 0.10] | 0.17 |
| control vs 3700 K | -0.16 | 0.85 | [-0.49, 0.17] | 0.34 |
| Climate chamber | -0.10 | 0.90 | [-0.38, 0.17] | 0.46 |

**Table S3.** Regression results (ANOVA table) for caterpillar body mass. The explanatory variables are ALAN treatment (three levels), Caterpillar instar (seven levels), climate chamber (two levels), and the interaction between treatment and instar.

Statistical levels of significance: *** < 0.001 ** < 0.01, * < 0.05

| **Bodymass** | | | | | |
| --- | --- | --- | --- | --- | --- |
| *Term* | *ChiSq* | *Df* | *p-value* | *R2* | *AIC* |
| (Intercept) | 201.11 | 1 | **< .001***** |  |  |
| ALAN treatment | 3.19 | 2 | 0.20 |  |  |
| Caterpillar instar | 1920.50 | 6 | **< .001***** |  |  |
| Climate chamber | 0.02 | 1 | 0.89 |  |  |
| treatment:instar | 15.05 | 12 | 0.24 | 0.93 | 557 |

**Table S4.** Pairwise comparison (post hoc test with FDR correction) of light treatments per larval stage to explain mean caterpillar body mass. The explanatory variables are ALAN treatments (3 levels) and the interactions between the treatments and larval instar (treatment:instar).

Statistical levels of significance: *** < 0.001 ** < 0.01, * < 0. 05.

| **Bodymass** | | | | | | |
| --- | --- | --- | --- | --- | --- | --- |
| *Contrasts* | *larvae instar* | *Difference* | *95% CI* | *df* | *t-ratio* | *p-value* |
| 2200 K - control | 2 | -0.11 | [-0.37, 0.16] | 248.21 | -0.95 | 0.55 |
| 2200 K - control | 3 | -0.01 | [-0.28, 0.26] | 248.21 | -0.09 | 0.93 |
| 2200 K - control | 4 | 0.10 | [-0.17, 0.37] | 248.21 | 0.91 | 0.48 |
| 2200 K - control | 5 | 0.11 | [-0.16, 0.38] | 248.21 | 1.00 | 0.48 |
| 2200 K - control | 6 | 0.03 | [-0.24, 0.31] | 255.54 | 0.31 | 0.76 |
| 2200 K - control | 7 | 0.05 | [-0.26, 0.36] | 341.95 | 0.37 | 0.71 |
| 2200 K - control | 8 | -0.16 | [-0.59, 0.27] | 477.27 | -0.90 | 0.37 |
| 3700 K - 2200 K | 2 | -0.01 | [-0.29, 0.28] | 245.72 | -0.05 | 0.96 |
| 3700 K - 2200 K | 3 | 0.19 | [-0.09, 0.47] | 245.72 | 1.61 | 0.23 |
| 3700 K - 2200 K | 4 | 0.08 | [-0.20, 0.37] | 245.72 | 0.70 | 0.48 |
| 3700 K - 2200 K | 5 | 0.03 | [-0.25, 0.32] | 245.72 | 0.28 | 0.78 |
| 3700 K - 2200 K | 6 | 0.07 | [-0.22, 0.35] | 247.99 | 0.56 | 0.76 |
| 3700 K - 2200 K | 7 | 0.15 | [-0.17, 0.47] | 333.93 | 1.11 | 0.40 |
| 3700 K - 2200 K | 8 | 0.48 | [ 0.03, 0.94] | 479.07 | 2.54 | **0.03*** |
| 3700 K - control | 2 | -0.11 | [-0.41, 0.19] | 246.51 | -0.90 | 0.55 |
| 3700 K - control | 3 | 0.18 | [-0.12, 0.48] | 246.51 | 1.44 | 0.23 |
| 3700 K - control | 4 | 0.18 | [-0.12, 0.48] | 246.51 | 1.47 | 0.43 |
| 3700 K - control | 5 | 0.14 | [-0.16, 0.44] | 246.51 | 1.16 | 0.48 |
| 3700 K - control | 6 | 0.10 | [-0.20, 0.40] | 250.42 | 0.81 | 0.76 |
| 3700 K - control | 7 | 0.20 | [-0.15, 0.54] | 335.24 | 1.38 | 0.40 |
| 3700 K - control | 8 | 0.32 | [-0.14, 0.79] | 469.97 | 1.69 | 0.14 |

**Table S5.** The number of caterpillars that pupated after a given number of instar (ranging from 5 to 10), shown seperately for each ALAN treatment (2200 K, 3700 K and the dark control).

|  | **Larval instar** | | | | | |
| --- | --- | --- | --- | --- | --- | --- |
| **Treatment** | **5** | **6** | **7** | **8** | **9** | **10** |
| 2200 K | 1 | 11 | 15 | 5 | 4 | 0 |
| 3700 K | 0 | 7 | 9 | 5 | 1 | 1 |
| control | 1 | 8 | 9 | 8 | 1 | 0 |

**Table S6.** Regression results (ANOVA table) for the mean final larval body mass. The explanatory variables are ALAN treatment (three levels), climate chamber (two levels), and the needed larval stages until pupation. Statistical levels of significance: *** < 0.001 ** < 0.01, * < 0.05

| **Final body mass** | | | | | |
| --- | --- | --- | --- | --- | --- |
| *Term* | *SumSq* | *Df* | *p-value* | *R2* | *AIC* |
| (Intercept) | 22.37 | 1 | **< .001***** |  |  |
| ALAN treatment | 0.67 | 2 | 0.14 |  |  |
| Climate chamber | 0.50 | 1 | 0.09 |  |  |
| larval stages | 5.38 | 1 | **< .001***** |  |  |
| Residuals | 13.48 | 81 |  | 0.34 | 97 |

**Table S7.** Pairwise comparison (Tukey post hoc test) of light treatments to explain mean final larval body mass. The explanatory variables are ALAN treatments (3 levels).

Statistical levels of significance: *** < 0.001 ** < 0.01, * < 0. 05.

| **Final body mass** | | | | |
| --- | --- | --- | --- | --- |
| *Contrasts* | *Difference* | *95% CI* | *t(81)* | *p-value* |
| 2200 K - 3700 K | -0.19 | [-0.45, 0.08] | -1.69 | 0.2 |
| 2200 K - control | 0.03 | [-0.22, 0.28] | 0.29 | 0.77 |
| 3700 K - control | 0.22 | [-0.07, 0.50] | 1.85 | 0.2 |

**Table S8.** Cox proportional hazard results for caterpillar pupation timing. The explanatory variables are ALAN treatment (three levels), climate chamber (two levels) and the needed larval stages until pupation. The model estimates hazard ratios based on treatment and chamber effects on pupation timing.

Statistical levels of significance: *** < 0.001; ** < 0.01; * < 0.05.

| *Term* | *Coefficent* | *Hazard ratio* | *95% CI* | *p-value* |
| --- | --- | --- | --- | --- |
| 2200 K vs 3700 K | 0.39 | 1.48 | [-0.20, 0.97] | 0.19 |
| control vs 3700 K | 0.13 | 1.14 | [-0.46, 0.72] | 0.67 |
| Climate chamber | -0.89 | 0.41 | [-1.39, -0.40] | **< .001***** |
| Larval stages | -1.44 | 0.24 | [-1.80, -1.8] | **< .001***** |

**Table S9.** Regression results (ANOVA table) for age of pupation. The explanatory variables are ALAN treatment (three levels), climate chamber (two levels), and the needed larval stages until pupation.

Statistical levels of significance: *** < 0.001 ** < 0.01, * < 0.05

| **Age of pupation** | | | | | |
| --- | --- | --- | --- | --- | --- |
| *Term* | *SumSq* | *Df* | *p-value* | *R2* | *AIC* |
| (Intercept) | 15.56 | 1 | **< .001***** |  |  |
| ALAN treatment | 0.01 | 2 | 0.54 |  |  |
| Climate chamber | 0.09 | 1 | **0.005**** |  |  |
| larval stages | 1.68 | 1 | **< .001***** |  |  |
| Residuals | 0.86 | 80 |  | 0.67 | -137 |

**Table S10.** Pairwise comparison (Tukey post hoc test) of light treatments to explain mean age of pupation. The explanatory variables are ALAN treatments (3 levels).

Statistical levels of significance: *** < 0.001 ** < 0.01, * < 0. 05.

| **Age of pupation** | | | | |
| --- | --- | --- | --- | --- |
| *Contrasts* | *Difference* | *95% CI* | *t(81)* | *p-value* |
| 2200 K - 3700 K | -0.06 | [-0.17, 0.06] | -1.15 | 0.76 |
| 2200 K - control | -0.02 | [-0.13, 0.09] | -0.42 | 0.95 |
| 3700 K - control | 0.04 | [-0.09, 0.16] | 0.72 | 0.95 |

**Table S11.** Regression results (ANOVA table) for pupal mass. The explanatory variables are ALAN treatment (three levels), climate chamber (two levels), and the needed larval stages until pupation.

Statistical levels of significance: *** < 0.001 ** < 0.01, * < 0.05

| **Pupal mass** | | | | | |
| --- | --- | --- | --- | --- | --- |
| *Term* | *SumSq* | *Df* | *p-value* | *R2* | *AIC* |
| (Intercept) | 29.12 | 1 | **< .001***** |  |  |
| ALAN treatment | 0.52 | 2 | 0.34 |  |  |
| Climate chamber | 0.47 | 1 | 0.17 |  |  |
| larval stages | 1.75 | 1 | **0.008**** |  |  |
| Residuals | 18.44 | 77 |  | 0.14 | 122 |

**Table S12.** Pairwise comparison (Tukey post hoc test) of light treatments to explain mean pupal mass. The explanatory variables are ALAN treatments (3 levels).

Statistical levels of significance: *** < 0.001 ** < 0.01, * < 0. 05.

| **Pupal mass** | | | | |
| --- | --- | --- | --- | --- |
| *Contrasts* | *Difference* | *95% CI* | *t(77)* | *p-value* |
| 2200 K - 3700 K | -0.14 | [-0.47, 0.19] | -1.06 | 0.58 |
| 2200 K - control | 0.07 | [-0.25, 0.38] | 0.52 | 0.61 |
| 3700 K - control | 0.21 | [-0.15, 0.57] | 1.44 | 0.46 |

**Table S13.** Regression results (ANOVA table) for pupal duration. The explanatory variables are ALAN treatment (three levels), climate chamber (two levels), and the needed larval stages until pupation.

Statistical levels of significance: *** < 0.001 ** < 0.01, * < 0.05

| **Pupal duration** | | | | | |
| --- | --- | --- | --- | --- | --- |
| *Term* | *SumSq* | *Df* | *p-value* | *R2* | *AIC* |
| (Intercept) | 5.37 | 1 | **< .001***** |  |  |
| ALAN treatment | 0.02 | 2 | 0.10 |  |  |
| Climate chamber | 0.01 | 1 | 0.19 |  |  |
| larval stages | 0.21 | 1 | **< .001***** |  |  |
| Residuals | 0.16 | 33 |  | 0.58 | -88 |

**Table S14.** Pairwise comparison (Tukey post hoc test) of light treatments to explain mean pupal duration. The explanatory variables are ALAN treatments (3 levels).

Statistical levels of significance: *** < 0.001 ** < 0.01, * < 0. 05.

| **Pupal duration** | | | | |
| --- | --- | --- | --- | --- |
| *Contrasts* | *Difference* | *95% CI* | *t(33)* | *p-value* |
| 2200 K - 3700 K | -0.02 | [-0.09, 0.06] | -0.62 | 0.54 |
| 2200 K - control | 0.05 | [-0.02, 0.12] | 1.65 | 0.22 |
| 3700 K - control | 0.06 | [-0.01, 0.14] | 2.13 | 0.12 |
